# Standardized bone marrow assessment, risk variables, and survival in dogs with myelodysplastic syndrome and acute myeloid leukemia

**DOI:** 10.1177/03009858241277982

**Published:** 2024-09-18

**Authors:** Anna M. Meredith, Janet Beeler-Marfisi, Olaf Berke, Anthony J. Mutsaers, Dorothee Bienzle

**Affiliations:** 1 University of Guelph, Guelph, ON, Canada

**Keywords:** canine, cytopenia, dysplasia, leukemia classification, myeloid neoplasia, myeloproliferative disease

## Abstract

Myelodysplastic syndrome (MDS) and acute myeloid leukemia (AML) are heterogeneous neoplasms of hematopoietic stem cells that are challenging to diagnose, differentiate, and prognosticate. Cytogenetic and mutational analyses are useful in humans but unavailable for dogs, where diagnosis and classification still rely largely on hematologic and morphologic assessment. The objectives of this study were to apply a classification scheme to myeloid neoplasms and to assess outcome in relation to predictor variables. Keyword search of a laboratory database, application of sequential exclusion criteria, and consensus from 3 reviewers yielded 70 cases of myeloid neoplasia with hematology results, and cytologic (11), histologic (14), or both (45) types of marrow specimens. Based on blast percentage and morphology, 42 cases were classified as MDS and 28 as AML. Dogs with MDS had significantly lower body weights, hemoglobin concentrations and blood blasts, and higher red blood cell size variability and platelet numbers than dogs with AML. Estimates of median survival using Kaplan-Meier curves for dogs with MDS and AML were 384 and 6 days, respectively (*P* < .001). The instantaneous risk of death for dogs with MDS was approximately 5× lower than that of dogs with AML. Significant predictor variables of survival were body weight, white blood cell count, platelet count, and percent blood blasts (*P* < .05). Hazard ratios (HRs) derived from best-fitting Cox regression models were 1.043, 0.998, and 1.061 for increased neutrophils, decreased platelets, and increased blood blasts, respectively. Findings from this study suggest that hematologic and morphologic variables are useful to predict outcomes in myeloid neoplasia.

Myelodysplastic syndrome (MDS) and acute myeloid leukemia (AML) are myeloid neoplasms that develop from nonlymphoid hematopoietic stem cells^4,24^ and are differential diagnoses in patients with at least 1 persistent cytopenia (anemia, thrombocytopenia, or neutropenia) on complete blood cell counts (CBCs). The basis of diagnosis is a sustained failure to produce an adequate number of red blood cells (RBCs), white blood cells (WBCs), or platelets, in combination with an increased proportion of blasts or dysplastic cells in bone marrow biopsy or postmortem specimens. Distinction of myeloid neoplasia from the effects of viral or vector-borne infections (eg, parvovirus, anaplasmosis), toxins (eg, phenobarbital), autoimmune responses, and dietary deficiencies can be challenging as these conditions may also result in transient dysplastic changes and cytopenias with or without increased blasts.^4,50,11,29^ Hence, diagnosis of myeloid neoplasia can be onerous and usually requires sequential CBCs and assessments of blood and marrow cell morphology combined with exclusion of non-neoplastic causes of cytopenia.

Classification of myeloid neoplasia into MDS or AML, and categorization and prognostication of entities within either category, in humans rest on the triad of lineage determination, clinical attributes, and biological features such as gene fusions, rearrangements, and mutations.^26,5^ It is recognized that clonal hematopoiesis (CH), which is a proliferation of somatically mutated progenitor cells, invariably precedes myeloid neoplasia but may be clinically silent over years.^53^ CH is increasingly frequent with progressive age and is associated with conditions such as cardiovascular and neurodegenerative diseases.^53^ If the mutation frequency in CH exceeds a specified threshold, and occurs in myeloid malignancy-associated genes, the term “clonal hematopoiesis of indeterminate potential” (CHIP) is applied. If CHIP is identified concurrent with cytopenia, the condition is termed “clonal cytopenia of undetermined significance.”^53^ Although recent work identified age-linked somatic mutations in CHIP-associated genes in geriatric dogs,^39^ and stable somatic mutations in leukocytes of older dogs,^27^ the significance of these findings remains to be further explored. Somatic mutations in nonhematopoietic genes recently identified in human patients with adult-onset severe autoinflammatory disease and MDS, such as the VEXAS (*v*acuoles, *E*1 enzyme, *X*-linked, *a*utoinflammatory, *s*omatic *UBA1* mutations) syndrome, link autoimmunity and hematopoietic disease, and further highlight the complexity of diagnosis and classification of myeloid neoplasia.^19^

According to the World Health Organization (WHO) classification of myeloid neoplasms in humans, MDS, recently termed “myelodysplastic syndrome/neoplasm” to emphasize the neoplastic nature (still abbreviated MDS), is broadly defined by cytopenia, dysplasia in >10% of cells of any lineage, and <20% blasts in marrow.^26^ Conversely, when diagnosed in the absence of specific genetic alterations, AML is defined by ≥20% blasts in blood or marrow. Relative to the fourth WHO classification (2016), more recent summaries of the forthcoming fifth edition emphasize genetic changes to a greater extent and morphologic features to a lesser extent.^4,26^ The contemporaneous International Consensus Classification presents a similar approach but with greater weight on morphology.^5,12^ In humans, MDS may be subcategorized by the presence of specific cytogenetic abnormalities and mutations and by the proportion of blasts. Multiple subcategories of AML characterized by specific genetic abnormalities are also recognized. Generally, MDS is a more indolent neoplasm than AML. Both MDS and AML may manifest with hypocellular, normocellular, or hypercellular marrow; MDS may evolve to AML, and AML may manifest with dysplasia. Hence, distinction between subtypes of myeloid neoplasia is challenging, and a continuum of disease has been proposed to encompass the extensive overlap.^3^ CH in patients without hematopoietic disease, and mutations in inflammatory genes in patients with MDS, illustrate the complex interconnectedness of hematopoiesis and immunity, and the challenge of accurate diagnosis and therapy.

In veterinary patients, MDS and AML are typically diagnosed and distinguished by similar clinical and morphologic criteria as in humans. Flow cytometric and cytochemical analysis to assist with lineage assignment and genetic analysis to identify mutations have been reported, but association with response to therapy and outcome remains undetermined.^32,20^ As in humans, there is a wide range of clinical and morphologic findings associated with hematopoietic disease, and while similar criteria to those in humans are applied, distinction of neoplastic disease from conditions responsive to immunosuppression or immunomodulation remains challenging. In general, dogs with AML are acutely ill, have marked cytopenia and variably cellular marrow while dogs with MDS have chronic vague illness, mild to marked persistent cytopenia, and normocellular to hypercellular marrow. Thresholds for the frequency of blasts and dysplastic cells similar to humans have been suggested.^35,2^

A grading scheme that correlates with outcome, using commonly available clinical, hematologic, and morphologic criteria, is needed for myeloid neoplasms in dogs. The goals of this study were to assess the survival of dogs diagnosed with a standardized scheme, derived from the WHO classification, as having MDS or AML and to assess the effect of predictor variables on survival. It was hypothesized that a diagnosis of MDS versus AML, a lower blast percentage in blood or marrow, and a milder degree of cytopenia would be associated with longer survival.

## Materials and Methods

### Case Selection

The database of the Animal Health Laboratory at the University of Guelph was searched for cases of MDS or AML between May 8, 2007 and June 6, 2018 using the terms “dysplas,” to capture the terms dysplasia or dysplastic, and “leukemia.” Inclusion criteria were availability of at least 1 CBC or a peripheral blood film (PBF); marrow evaluation consisting of at least 1 marrow aspirate or core impression smear (bone marrow aspirate [BMA]), core biopsy (bone marrow core [BMC]), or bone marrow postmortem (BMPM) section; and survival data. Cases also included those with suspected immune-mediated disease that was refractory to treatment. Cases were excluded if (1) there was not at least 1 marrow preparation of adequate quality, defined as BMA preparation of adequate cellularity to differentially count 500 cells, BMC section with at least 3 intertrabecular spaces free of artifact, or BMPM section free of marked autolysis;^45^ (2) lymphocytic leukemia had been diagnosed by flow cytometric or immunohistochemical (IHC) analysis, or occurred subsequent to a previous diagnosis of lymphoma;^10,28^ (3) therapy with drugs known to cause myelodysplasia or another cause of cytopenia or myelodysplasia was identified (including immune-mediated diseases, inflammation, and iron deficiency);^50,11^ (4) a nonhematopoietic neoplasm affected the marrow; or (5) survival could not be determined (Supplemental Figure S1).

Patient demographic information, including age, sex, weight, breed, date of diagnosis, type of treatment, and date and cause of death were obtained from medical records or phone consultation with referring veterinarians or owners. Treatment was categorized as supportive, immunosuppressive, or chemotherapeutic, ie, employing a single or combination of cytotoxic agents. In 3 purebred patients, weight was not recorded, so the average breed weight by sex from the American Kennel Club was assigned.^41^ One case had a PBF but no automated hematology analyzer results; hence, numbers were estimated from review of the blood film following Animal Health Laboratory standard operating protocols.

The use of archived samples was confirmed as exempt from requiring Institutional Animal Care Committee approval.

### Case Assessment

**Figure 1.**
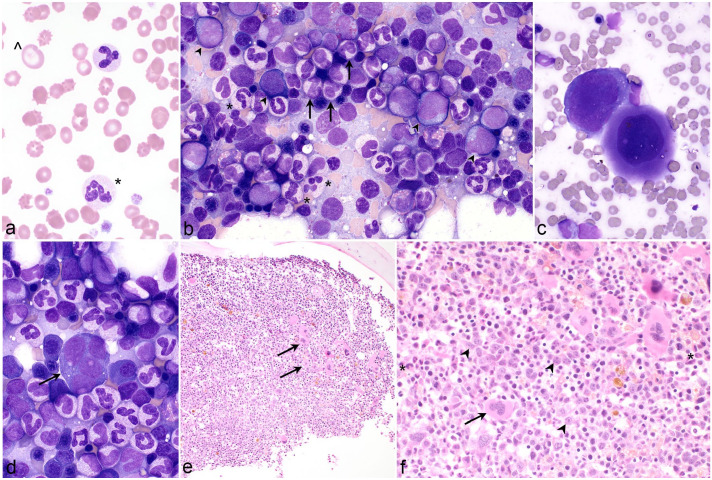
Myelodysplastic syndrome, dog. (a) Blood film with dysplastic neutrophils (*) that have unevenly sized nuclear lobes and giant red blood cells (^) reflecting increased red cell distribution width. Case 1. Wright stain (WS). (b) The marrow aspirate is highly cellular and has a predominance of granulocytes. There are approximately 10% blasts (arrowheads). Of total, 15% of segmented neutrophils have dysplastic changes (*), and there are dysplastic band neutrophils (arrows). Case 2. WS. (c) An immature megakaryocyte with abnormal nuclear lobulation (left) and an abnormally small megakaryocyte with mature cytoplasm and a hypolobulated nucleus (right). Case 2. WS. (d) A micromegakaryocyte, ~24 µm in diameter with multiple, widely separated nuclei (arrow). Case 2. WS. (e) The marrow core is hypercellular despite chronic marked anemia, and megakaryocytes are clustered (arrows). Case 3. Hematoxylin and eosin (HE). (f) Higher magnification of (e) illustrating an increased proportion of blasts (arrowheads) in the center of the intertrabecular space; a megakaryocyte with abnormal nuclear lobulation, open chromatin, and nucleoli (arrow); and dysplastic giant band neutrophils (*). Case 3. HE.

**Figure 2.**
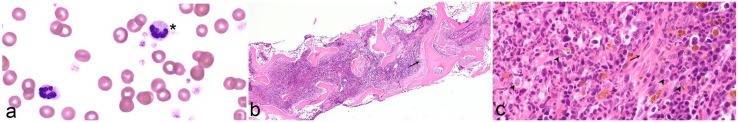
Myelodysplastic syndrome, dog. (a) The blood film has dysplastic neutrophils (*) characterized by incomplete and uneven nuclear segmentation. Note also frequent platelets. The dog had chronic nonregenerative anemia, a normal leukocyte count, and thrombocytosis (554 × 10^9^/L; reference interval 117-418 × 10^9^/L). The marrow aspirate had no particles. Case 4. Wright stain. (b) The marrow core is nearly devoid of adipocytes is densely cellular with paratrabecular fibrosis (arrow). Case 5. Hematoxylin and eosin (HE). (c) At higher magnification, there are slender bundles of collagen interspersed among hematopoietic cells (arrow). Blasts comprise approximately 5% of cells (arrowheads). Case 5. HE.

**Figure 3.**
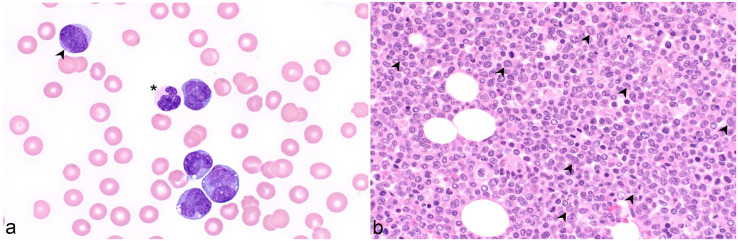
Acute myeloid leukemia, dog. Case 6. (a) The blood film contains occasional blasts (arrowhead), numerous large myelomonocytes with irregular nuclear shapes, and occasional cytoplasmic vacuoles. Neutrophils are dysplastic with irregular nuclear lobes and incomplete segmentation (*). The dog had a leukocytosis of 157 × 10^9^/L (reference interval = 4.9-15.4 × 10^9^/L), thrombocytopenia, and anemia. Wright stain. (b) The postmortem marrow section is nearly 100% cellular and includes many cells with irregular nuclear shapes and large, sometimes amphophilic, nucleoli (arrowheads). Hematoxylin and eosin.

**Figure 4.**
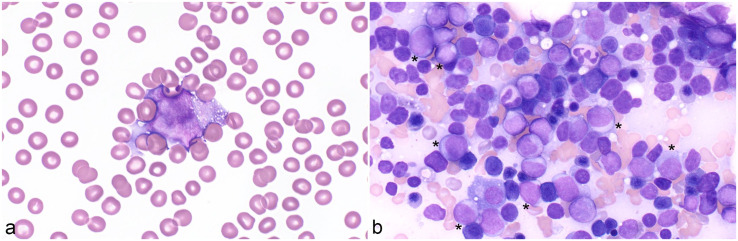
Acute myeloid leukemia, dog. Case 7. Wright stain. (a) The dog had neutropenia, anemia, and thrombocytopenia. Approximately 40% of leukocytes are large blasts. (b) The marrow aspirate is highly cellular and contains approximately 45% blasts (*).

Three assessors, blinded to case data other than CBC numerical results, examined PBF and marrow slides and generated de novo independent interpretations for each case using standardized assessment forms adapted from the WHO guidelines (Supplemental Tables S1 and S2).^4,1,40,42,43,9^ For PBF, 1 assessor performed a 200-cell differential count, and all assessors estimated blast percentage and assessed cells for morphologic features such as dysplasia and neutrophil toxicity. Blasts were identified by scant basophilic cytoplasm, a high nucleocytoplasmic ratio, fine chromatin pattern, and nucleoli (Figs. 1–4).^4,34^ Dysplasia was best appreciated in BMA, but some features could also be observed in BMC and BMPM. In megakaryocytes, hypolobulated nuclei (Fig. 1c), micromegakaryocytes (Fig. 1d), location near trabeculae, and clustering (Fig. 1e) were considered. In the erythroid series megaloblasts, nucleocytoplasmic asynchrony, multinucleation, and bilobed or irregularly contoured nuclei were noted. Granulocyte dysplasia could include giant metamyelocyte or band neutrophils (Figs. 1e, 2a, and 3a) and partial or variably sized nuclear lobulation in segmented neutrophils (Figs. 1a–b, 2a, and 3a).^5,13,9^ For marrow samples, 1 assessor performed a 500-cell differential count on the BMA slide and determined the percent dysplastic erythroid, granulocytic, and megakaryocytic cells. In samples with >50% blasts, only 300 cells were differentially counted.^40,9^ All assessors categorized cases as MDS, AML, or “other,” with the latter encompassing nonmyeloid neoplasms and non-neoplastic conditions. Once independent evaluations were complete, the history, prior diagnosis, treatment, duration of cytopenia(s), hematologic progression, survival, and, where available, postmortem findings were added to the data set. The assessors then jointly reviewed all cases taking all data into consideration to confirm or revise their initial diagnosis and to arrive at a consensus in cases with discordant interpretations.

### Data Analysis

Patient characteristics and laboratory findings were summarized using standard descriptive statistics including sample size, mean, standard deviation, and range. In addition, the distributions of patient characteristics and laboratory findings in dogs with MDS and AML were further assessed using 95% confidence intervals (CI_95%_), *t*-tests, and Fisher’s exact test. Survival was measured in days and calculated as the time from diagnosis to death. Patients still alive at the time of last follow-up were censored from the analysis.

The Kaplan-Meier survival analysis, log-rank test, and Cox proportional hazards regression modeling were performed to estimate the survival of patients with MDS compared with AML and to determine HRs, respectively.^25,47^ A Cox regression model was used to assess the utility of risk factors for predicting death in dogs with MDS or AML.^18,4,15,47^

Survival was estimated using a Cox proportional hazards model:^47^

$$h_{i}\left(t\right)=h_{0}\left(t\right)exp\left(\left\{X_{i}\beta \right\}\right)$$

where *i* = 1,. . ., *n* is an index identifying the *n* = 70 dogs in the sample, and *h*() is the hazard function at time *t*. Specifically, *h_i_* is the hazard, or instantaneous risk (ie, the probability of death at a specific point in time, given that the patient has survived up to that moment, hereafter “risk of death”), of dog *i* dying, and *h*_0_(*t*) is a time-dependent baseline hazard. The effect of likely risk factors, *X_i_*, on survival was measured by the linear combination {*X*_i_β}, where *X*_i_ is comprised of the *i*th dog’s characteristics, and β measures the effect of *X* on survival, and was interpreted as (*HR*) = *exp*(β). Typically, exp(β) was interpreted as follows: for every 1-unit increase in *X*, the hazard changes by exp(β). However, to arrive at a clinically relevant interpretation, it was sometimes necessary to examine the effects of an increase by, eg, *k* units in the risk factor, which can be calculated from *exp*(k•β) with a CI_95%_ (HR) = exp(k[β ± 1.96•*SE*(β)]).

Variables reported to influence survival in humans with MDS or AML (body weight, red cell distribution width, nucleated RBC, WBC, neutrophils, platelets, blast percentage in peripheral blood, and treatment category) were individually assessed. To estimate HRs associated with potential death risk factors, and to compute the corresponding CI_95%_, Cox regression modeling was applied using backward selection by optimizing the Akaike information criterion. Various model diagnostic tests were employed to identify violations of model assumptions. The proportional hazards assumption of the Cox regression model was assessed by inspecting a scatterplot of scaled Schoenfeld residuals against time, as well as the graphic result from the Grambsch-Therneau test. The linearity assumption was assessed using a scatterplot of martingale residuals. Finally, interactions among variables were investigated within the model to identify possible confounding or collinear variables that were then eliminated from the model.

Data analyses were performed using R and RStudio.^46^ A significance level of α = 0.05 was applied. With regard to hypothesis generation, significance was considered as “exploratory” rather than as “confirmatory.”^30^

## Results

### Cases

The database search yielded 237 cases containing the specific keywords. This number was reduced to 81 through sequential application of exclusion criteria (Supplemental Figure S1). Samples from these 81 cases were then scored by 3 pathologists blinded to all case data except for CBC numerical data yielding a consensus interpretation of MDS in 42 cases, AML in 28 cases, and “other” in 11 cases (Supplemental Figure S1). Cases with an interpretation other than myeloid neoplasia were omitted from subsequent analysis. Of cases with MDS, the most common sample types were BMA and BMC (*n* = 26); followed by BMA (*n* = 6); BMC (*n* = 4); BMA and BMPM (*n* = 4); and BMA, BMC, and BMPM (*n* = 2). Cases diagnosed as AML most commonly had BMPM (*n* = 10) samples; BMA and BMC (*n* = 6); BMA (*n* = 5), BMA, and BMPM (*n* = 4); and BMA, BMC, and BMPM (*n* = 3) (Supplemental Figure S1). Of the 4 cases of MDS with only a BMC, the interpretation was based on cellularity, estimated blast percentage, megakaryocyte clustering (Fig. 1e), and giant bands (Fig. 1f).

### Patient Characteristics and Hematologic Findings

Patient demographic information, hematologic findings, and descriptive statistics are summarized in Table 1 and presented in Supplemental Tables S5 and S6. Dogs with MDS had significantly lower body weights, lower hemoglobin, higher red cell distribution widths, higher platelet counts, and lower blast percentages in peripheral blood than dogs with AML (Table 1). Forty of the 42 (95%) dogs with MDS were anemic. Thirteen of these 42 (31%) dogs were leukopenic due to neutropenia in 10 of 42 dogs (24%). Seven of 10 neutropenic dogs were also thrombocytopenic. Thirty-two of the 42 (76%) dogs with MDS had no blasts in blood (Figs. 1 and 2 and Supplemental Table S5). Leukocytosis due to neutrophilia was present in 6 of the 42 (14%) dogs with MDS.

**Table 1.** Patient characteristics and laboratory findings in dogs with myelodysplastic syndrome (MDS, *n* = 42) or acute myeloid leukemia (AML, *n* = 28).

	MDS		AML		Confidence interval (95%)^a^	*P*
	Mean ± (SD)	Range	Mean ± (SD)	Range	AML—MDS	*t*-test^b^
Age (years)	7.8 ± (2.6)	3.4-15.0	7.2 ± (3.2)	1.1-12	-2.099, 0.894	.423
Weight (kg)	17.3 ± (11.7)	4.1-48.8	23.8 ± (13.2)	2.0-49.7	0.231, 12.764	**.042**
Sex	Intact	Altered	Intact	Altered		
Male, n (%)	2 (4.8)	12 (28.6)	3 (10.7)	8 (28.6)		
Female, n (%)	2 (4.8)	26 (61.9)	1 (3.6)	16 (57.1)		
Hematocrit (L/L)	0.21 ± (0.06)	0.07-0.44	0.26 ± (0.1)	0.15-0.48	0.011, 0.100	.099
Hemoglobin (g/L)	66.8 ± (31.1)	22-146	82.2 ± (29.6)	50-163	0.433, 30.244	**.044**
MCV (fL)	73.0 ± (8.4)	57-98	74.3 ± (4.8)	66-88	-1.949, 4.563	.426
RDW	16.5 ± (3.9)	12.6-27.5	14.9 ± (2.6)	11.6-19.7	-3.102, -0.116	**.035**
WBC (×10^9^/L)	9.4 ± (8.4)	0.4-36.4	20.0 ± (31.2)	1.4-157.7	-1.850, 22.993	.092
Neutrophils (×10^9^/L)	6.8 ± (6.5)	0-26.1	9.0 ± (9.0)	0.01-38.8	-2.744, 6.973	.384
Lymphocytes (×10^9^/L)	1.0 ± (0.6)	0.1-2.5	2.0 ± (3.2)	0.3-12.6	-0.175, 2.256	.090
Monocytes (×10^9^/L)	1.2 ± (1.3)	0-7.5	4.8 ± (14.3)	0-73.9	-1.871, 9.026	.189
Platelets (×10^9^/L)	251 ± (213.9)	7-948	96 ± (79.4)	5-366	-227.507, -81.351	.001
Blast % blood	0.19 ± (0.4)	0-1.5	8.8 ± (12.7)	0-42.5	3.609, 13.532	**.001**

Means of each group were compared. Significance differences (*P* < .05) between dogs with MDS and AML are bolded.

Abbreviations: MCV, mean corpuscular volume; n, number of cases; RDW, red cell distribution width; SD standard deviation; WBC, white blood cell count.

a Confidence interval estimates the magnitude of the difference between groups, ie, AML vs MDS.

b *P-*value calculated using Welch’s test.

Of dogs with AML, 24 of 28 (85%) were anemic, 9 of 28 (32%) were leukopenic caused by neutropenia, but overall, 13 of 28 (46%) were neutropenic. In this group, 19 of 28 (68%) dogs were thrombocytopenic, and 5 of 28 (18%) were pancytopenia. Leukocytosis was observed in 10 of 28 (36%) dogs with AML and was caused by neutrophilia in 7 of 28 (25%) or by neutrophilia and increased blasts in 5 of 28 (18%) dogs. Dogs with AML had a mean of 8.8% blasts and 7 of 28 (25%) had >20% blasts in blood (Figs. 3 and 4 and Supplemental Table S6).

Eight of 27 BMA samples from dogs with MDS were of limited cellularity. The BMC from each of these 8 dogs had evidence of myelofibrosis. All 18 BMA samples from dogs with AML were 71% to 100% cellular.

Dogs with MDS were most commonly of mixed breed (*n* = 12, 29%), followed by wheaten terrier (*n* = 6, 14%), Shih Tzu (*n* = 4, 10%), Labrador retriever (*n* = 3, 7%), dachshund (*n* = 2, 5%), and Boston terrier (n = 2, 5%). There were 1 each of American cocker spaniel, beagle, boxer, cairn terrier, German shepherd, lurcher, miniature dachshund, miniature schnauzer, old english sheepdog, standard poodle, Shetland sheepdog, toy fox terrier, and toy poodle in the MDS group. The most frequent breeds among dogs with AML were mixed breed (*n* = 9, 32%), golden retriever (*n* = 4, 14%), German shepherd (*n* = 2, 7%), and vizsla (*n* = 2, 7%). The AML group contained 1 each of Australian shepherd, Chinese crested, Chihuahua, doberman pinscher, English bulldog, miniature schnauzer, rottweiler, standard poodle, Shetland sheepdog, Shih Tzu, and whippet.

### Survival and Treatment

**Figure 5.**
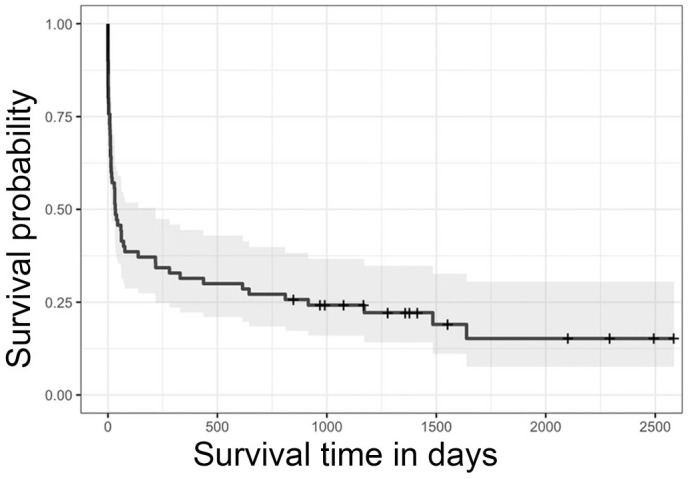
Kaplan-Meier survival curve for 70 dogs diagnosed with myelodysplastic syndrome or acute myeloid leukemia. The shaded area represents the 95% confidence interval. Hatch marks indicate the censored data.

**Figure 6.**
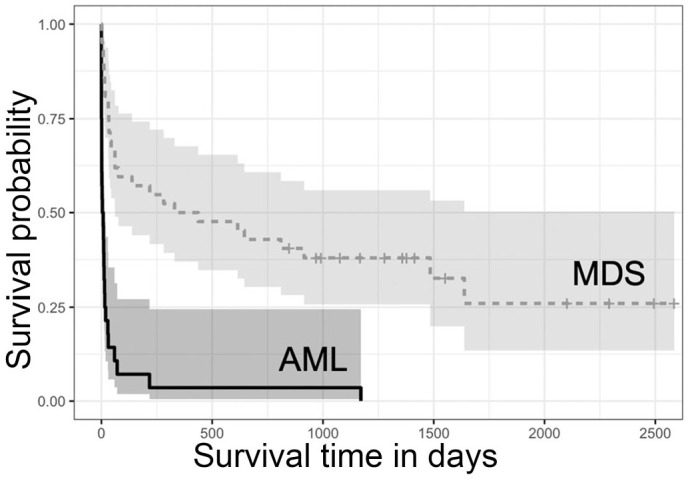
Kaplan-Meier survival curves for 70 dogs by category of myeloid neoplasm; myelodysplastic syndrome (MDS; *n* = 42, dotted line), acute myeloid leukemia (AML; *n* = 28, solid line). Dogs diagnosed with MDS survived longer than dogs diagnosed with AML (*P* < .001). Shaded areas represent the 95% confidence intervals of each curve; hatch marks reflect censored data.

Fifty-six of 70 (80%) dogs died during the study period with a median survival of 33.5 days (CI_95%_ = [15, 217]). The maximum survival was 2586 days for 1 dog with MDS (Fig. 5). The 42 dogs with MDS had a median survival of 384 days (CI_95%_ = [61, >2586]). Twenty-eight (67%) dogs with MDS died during the study period; the 14 still alive at final follow-up were censored in the survival analysis (Fig. 6). The 28 dogs with AML had a median survival of 6 days (CI_95%_ = [1, 15]) and all died within the study period. The log-rank test between dogs with MDS and AML signaled significantly longer survival for dogs with MDS (*P* < .001). The difference in survival between dogs with MDS and AML was further quantified using a Cox model fitted with “final case diagnosis” as the only predictor variable. The marginal HR for death in dogs with MDS compared to those with AML was 0.18 (CI_95%_ = [0.10, 0.33], *P* < .001), indicating that the risk of death for dog with MDS was approximately 5 times lower than that of dogs with AML. To explore the presence of an association between sex and intactness of dogs with the health outcome (MDS vs AML), the Fisher’s exact test was applied with nonconclusive results. There was insufficient evidence for an association between health status and the sex of intact dogs or the biological sex of intact and altered dogs with their health status (*P* = .70 and *P* = .62, respectively).

The majority of dogs with MDS and AML received only immunosuppressive therapy (MDS *n* = 31, 74%; AML *n* = 15, 54%). The most common treatment was glucocorticoids with some instances of cyclosporine or azathioprine. Fewer patients received chemotherapy (MDS *n* = 7, 17%; AML *n* = 10, 36%) or supportive therapy only (MDS *n* = 4, 10%; AML *n* = 3, 11%). Dogs with MDS or AML that were neutropenic and received chemotherapy had the longest mean survival. Among dogs with MDS, 2 with neutropenia that received multiple doses of chemotherapy and 1 that received 1 dose of L-asparaginase lived beyond the end of the study period. In dogs with AML, 2 with neutropenia received multiple cycles of doxorubicin and cytarabine and survived 217 and 1171 days, respectively.

Three dogs initially diagnosed with MDS, based on BMA blasts <20%, were subsequently (within 2 weeks) diagnosed with AML on BMPM. One day was an 8-year-old, female spayed, mixed-breed dog of 48 kg, and 1 dog was an 11-year-old Chihuahua of 2 kg. The third dog was a 7.5-year-old golden retriever of 39 kg. On initial CBC and PBF evaluations, the first dog had a moderate nonregenerative anemia, leukocytosis, neutrophilia, and platelets within the reference interval. The second dog had a marked regenerative anemia, WBC and neutrophils within the reference interval, and thrombocytopenia. The third dog had a hematocrit within the reference interval, leukopenia, neutropenia, and thrombocytopenia. None had blasts on the PBF. The first dog survived 10 days, the second dog survived 4 days, and the third dog survived 12 days from the initial diagnosis.

### Single Variable Analysis

Descriptive statistics and estimates of the HR from a Cox proportional hazards model for individual predictor variables using data from all 70 dogs are summarized in Table 2. Significant predictors of survival were body weight (*P* = .021), WBC (*P* = .003), platelet count (*P* = .021), and blast percentage in peripheral blood (*P* < .001).

**Table 2.** Single variable analysis using a Cox regression model in 70 dogs with a diagnosis of myelodysplastic syndrome (MDS) or acute myeloid leukemia (AML).

Variable	Minimum	Median	Maximum	HR^a^	Confidence interval (95%)	*P* ^b^
Weight (kg)	2	16	50	1.023	1.003, 1.043	**.021**
Hematocrit (L/L)	0.07	0.21	0.48	1.537	0.094, 25.070	.763
WBC (×10^9^/L)	0.4	7.2	157.7	1.017	1.005, 1.028	**.003**
Neutrophils (10^9^/L)	0.0	4.3	38.8	1.025	0.991, 1.060	.159
Platelets (×10^9^/L)	5	128	948	0.998	0.996, 1.000	**.021**
Blast % PBF	0.0	0.0	42.5	1.065	1.034, 1.096	**<.001**
Blast % BMA						.300
Reference 0 = <5						
1 = 5-9				1.979	0.599, 6.538	
2 = 10-19				2.436	0.739, 8.028	
3 = >20				2.729	0.976, 7.634	
Blast % BMC						.300
Reference 0 = <5						
1 = 5-9				3.680	0.466, 29.060	
2 = 10-19				2.645	0.331, 21.170	
3 = >20				5.299	0.632, 44.450	

Significance is set at *P* < .05.

Abbreviations: BMA, bone marrow aspirate (*n* = 38), < 5% was used as reference category; BMC, bone marrow core biopsy (*n* = 40), < 5% was used as reference category; HR, hazard ratio; PBF, peripheral blood film; WBC, white blood cell count.

a Hazard ratio, the instantaneous risk of death per unit increase or decrease in the variable.

b Wald or likelihood ratio tests for continuous and categorical predictors, respectively.

Bolded values represent the statistical significance.

### Model Selection of Predictors

The best-fitting Cox model to explain survival was:

$$h\left(t\right)=\;h_{0}\left(t\right)\;*\;exp\left(\mathrm{platelets}*\beta_{1}+\;\mathrm{neutrophils}*\beta_{2}+\;\mathrm{blast}\;\%\;\mathrm{in}\;\mathrm{PBF}*\beta_{3}\right)$$

These variables were all significant at the 5% level, and results from the model are summarized in Table 3. Model diagnostic tests did not indicate a violation of model assumptions. Although the HRs from the model correspond to a 1-unit increase in each predictor variable, more clinically meaningful interpretations can be obtained by considering larger changes. For example, a decrease in platelet count of 50 × 10^9^/L yields an HR of exp(−50·β) = 1.10, CI_95%_(HR) = [1.01, 1.20], indicating a 10% increased risk of death. If the neutrophil count increases by 5 ×10^9^/L, the HR is 1.24, CI_95%_(HR) = [1.05, 1.46], indicating a 24% increase in the risk of death. The HR for a 2% increase in peripheral blood blast percentage is 1.13, CI_95%_(HR) = [1.06, 1.20], and for a 10% increase in blasts is 1.80, CI_95%_(HR) = [1.33, 2.44], representing a 13% and 80% increased risk of death, respectively.

**Table 3.** Hematologic variables that best predict survival.

Predictor variable	Coefficient	Standard error of coefficient	Hazard ratio (HR)	Confidence interval for HR	*P* ^a^
Neutrophils (×10^9^/L)	0.0423	0.0168	1.043	1.009, 1.078	**.011**
Platelets (×10^9^/L)	-0.0019	0.0008	0.998	0.996, 0.999	**.023**
Blast % on PBF	0.0589	0.0155	1.061	1.029, 1.093	**<.001**

A best-fitting Cox regression model was used for analysis, based on 70 dogs with a diagnosis of myelodysplastic syndrome (MDS) or acute myeloid leukemia (AML), where 56 deaths occurred. Significance level α = 0.05. Abbreviation: PBF, peripheral blood film.

a *P-*values refer to Wald tests.

Bolded values indicate statistical significance.

## Discussion

This is the first comprehensive study to evaluate outcome in dogs with myeloid neoplasia and to compare survival between dogs with MDS and AML diagnosed using a standardized classification scheme. Dogs with MDS had a much lower risk of death and much longer survival than those with AML, which agrees with previous case reports.^31,33,51,14,8,38^ The choice to include and compare both MDS and AML was deliberate because these neoplasms exist on a spectrum both clinically and diagnostically, and criteria for distinguishing them are ill defined in animals.^24,38^

Across both disease categories, dogs with MDS were more likely to weigh less, have lower hemoglobin concentrations, a higher red cell distribution width, and fewer blasts in blood than dogs with AML. Dogs with higher weights were more likely to have AML and decreased survival, suggesting that larger dogs are more prone to AML. The average weight in dogs with AML was lower than previously reported, likely reflecting the lack of giant breed dogs in this study.^8^ This is the first study with complete demographic data for MDS, precluding direct comparisons of body weight with previous works; however, in 2 case reports of 3 dogs, 2 were small breed.^14,21^ In another case study, it was surmised that of 19 miniature dachshunds with severe nonregenerative anemia, 10 likely had MDS.^44^ Although the number of wheaten terriers appears high, a relative breed-associated risk could not be determined within the confines of this study.

A higher red cell distribution width in dogs with MDS than AML likely reflects prolonged abnormal erythropoiesis yielding RBC of highly variable size, similar to humans with MDS.^48^ The association between WBC and survival reflected the trend for dogs with MDS to have a lower mean WBC compared to dogs with AML. This is similar to previous studies where the majority of dogs with AML had a normal to increased WBC.^49,8,35^ The finding is also consistent with MDS being a disease characterized by cytopenia due to ineffective hematopoiesis and progressive but gradual clonal expansion of neoplastic cells, whereas AML is a neoplasm with greater proliferation of hematopoietic precursors and is more likely to manifest with leukocytosis and blasts rather than leukopenia.^4^

Direct comparisons with previous studies of WBC, neutrophil, blast, and platelet counts are limited due to differences in case data presentations and analyses.^49,8,33,35^ Although patterns in mean values were similar to previous reports,^8^,^33^,^35^ some findings differed. Namely, in this study’s AML cases, leukocytosis and leukopenia were almost equally frequent. Leukocytosis was often accompanied by neutrophilia and increased blasts in peripheral blood, rather than neutropenia as noted in previous reports^49,35^ (Supplemental Table S6).

The blast percentage in peripheral blood was an expected predictor variable as frequent blasts suggest advanced disease in both MDS and AML (Table 2). Decreased platelet count was associated with an increase in the risk of death, because dogs with AML tended to have lower platelet counts than dogs with MDS, (Table 2), mirroring previous reports in dogs and humans.^23,31,51,14,21,49,8,38,16,36,6,44,35^

The Cox regression model to predict the risk of death after a diagnosis with MDS or AML included neutrophils, platelets, and blood blast percentages, and can be used to predict patient survival (Table 3). Platelets and blast percentages in peripheral blood were expected predictors, but increased neutrophil count as a predictor of death was unexpected. Generally, in humans with MDS and AML, the degree of neutropenia is a negative prognostic indicator.^7,22,36^ However, in this study, it was noted that neutropenic patients that received chemotherapy had the longest survival, including 2 dogs with AML with prolonged survival that received the most aggressive chemotherapy protocols. Although the potential effect of treatment interactions was evaluated in the model selection, it is possible that these cases skewed the relationship between the HR of death and neutrophil count. It is also conceivable that neutropenic dogs were treated more aggressively, or that the finding was coincidental.

Three cases of transformation from MDS to AML were suspected. In humans, the rate of transformation ranges from 5% to 50%^17^ but neither the incidence nor the time to transformation is known in canines.

The general limitations of all retrospective studies apply to this study. Case numbers were limited, although higher than previous studies. The study period spanned 11 years, and the search terms were selected to capture as many cases as possible despite evolving nomenclature, but it is likely that some cases of myeloid neoplasia were not coded as such. Furthermore, many dogs received some sort of immunosuppressive treatment prior to marrow assessment, most often glucocorticoids but also sometimes cyclosporine or azathioprine; only those that did not have evidence of hematopoietic recovery were retained in the study. Dysplasia and subsequent transformation to MDS was reported in a person with protracted azathioprine administration.^52^ However, although drug-associated dysplasia has been reported in dogs,^50^ transformation to drug-associated MDS has not. Ideally, marrow evaluation should be performed prior to any treatment to minimize the confounding effects of drug administration and repeat marrow assessments should be undertaken after cessation of the drug. Notwithstanding, our exclusion criteria aimed to remove all cases of secondary myelodysplasia, which may have also removed some cases with actual MDS. In some previous studies, immunophenotyping was used for the diagnosis of AML and other leukemias.^49,8,35,38^ Using an appropriate panel of antibodies and analysis, immunophenotyping is helpful to distinguish lymphoid blasts from myeloid blasts, but is neither widely available nor standardized, and of limited utility on its own to distinguish different types of myeloid neoplasia.^37^

In the absence of genetic and molecular tests, it is more challenging to diagnose and predict the outcome of myeloid neoplasia in dogs relative to humans. Atypical inflammatory responses, which may include the condition precursor-targeted immune-mediated anemia, may precede or be part of MDS, and account for response to immunosuppressive therapy in some cases.

Although this study represents a step forward in correlating routine laboratory findings in different types of myeloid neoplasia with outcome, a greater number of cases, standardized sample acquisition and analysis, consistent implementation of therapy, and analysis of quality of life would strengthen these preliminary findings. We propose the use of clinical, hematologic, and morphologic criteria for prognostically meaningful distinction of MDS and AML until incorporation of genetic data will allow more refined classification of myeloid neoplasms in dogs.

## Supplemental Material

sj-pdf-1-vet-10.1177_03009858241277982 – Supplemental material for Standardized bone marrow assessment, risk variables, and survival in dogs with myelodysplastic syndrome and acute myeloid leukemia Supplemental material, sj-pdf-1-vet-10.1177_03009858241277982 for Standardized bone marrow assessment, risk variables, and survival in dogs with myelodysplastic syndrome and acute myeloid leukemia by Anna M. Meredith, Janet Beeler-Marfisi, Olaf Berke, Anthony J. Mutsaers and Dorothee Bienzle in Veterinary Pathology
