# Supplementary material for: Standardized bone marrow assessment, risk variables, and survival in dogs with myelodysplastic syndrome and acute myeloid leukemia
Source: Vet Pathol. 2024 Sep 18;62(1):64–73. doi: 10.1177/03009858241277982 (PMC11697499; doi:10.1177/03009858241277982)
Supplement: sj-pdf-1-vet-10.1177_03009858241277982 – Supplemental material for Standardized bone marrow assessment, risk variables, and survival in dogs with myelodysplastic syndrome and acute myeloid leukemia [file sj-pdf-1-vet-10.1177_03009858241277982.pdf]

Supplemental Materials

Standardized bone marrow assessment, risk variables, and survival in dogs with myelodysplastic syndrome and acute myeloid leukemia

Anna M. Meredith, Janet Beeler-Marfisi, Olaf Berke, Anthony J. Mutsaers, Dorothee Bienzle

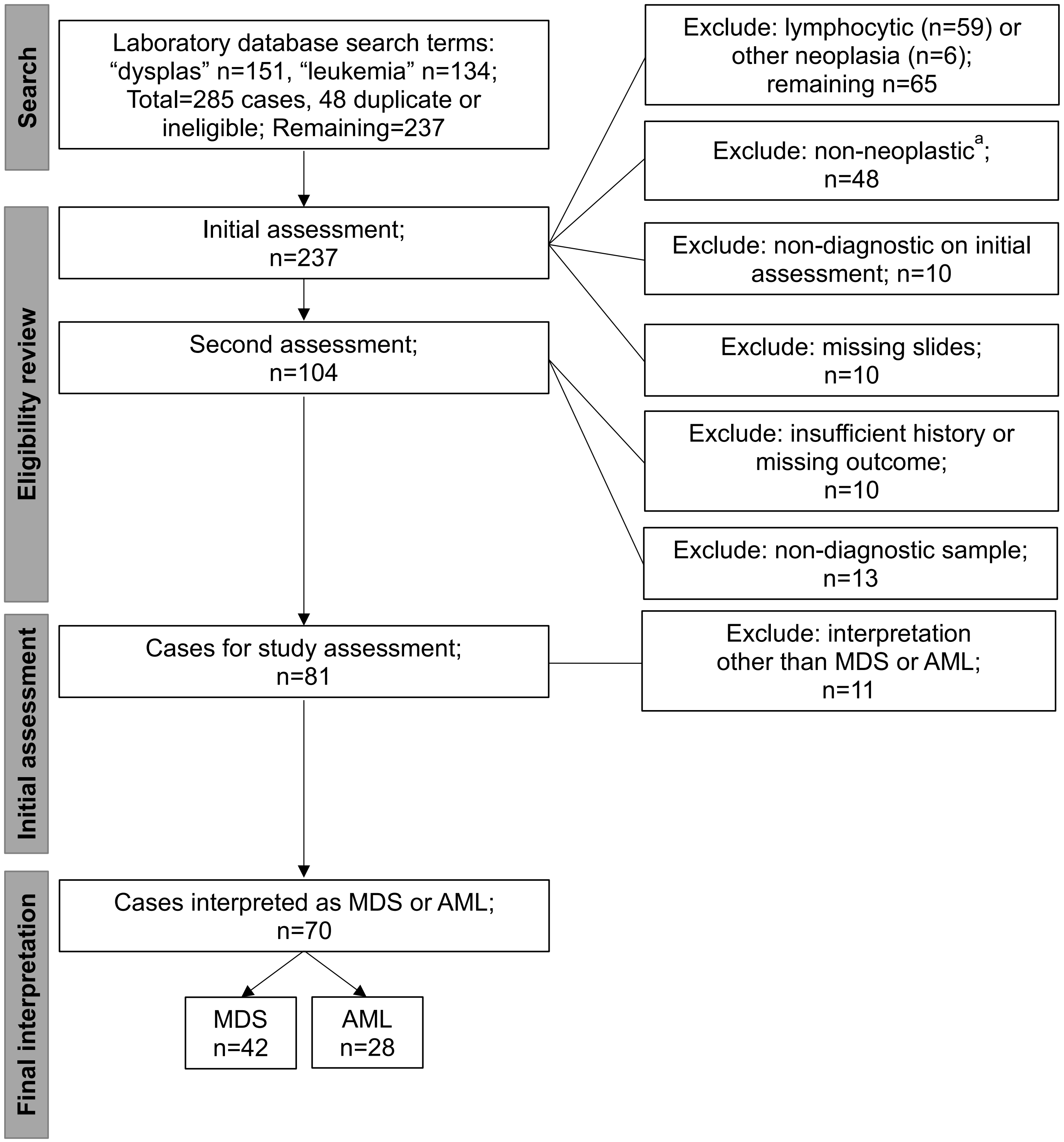

Supplemental Figure S1. Flowchart of database search, case selection, interpretation, and breakdown of the types and number of marrow samples analyzed. The search term "dysplas" was used to identify either "dysplasia" or "dysplastic" within the record. MDS, myelodysplastic syndrome; AML, acute myeloid leukemia; BMA, bone marrow aspirate; BMC, bone marrow core biopsy; BMPM, bone marrow postmortem marrow section.

[a]Immune-mediated disease, therapy-associated myelodysplasia, systemic inflammation, or iron deficiency.

Supplemental Table S1. Scoring of peripheral blood film variables.

**Blasts**<br>(200-cell differential count)	0 = none<br>1 = 1-4%<br>2 = 5-19%<br>3 = >19%	**Neutrophil toxicity**	0 = absent<br>1 = mild<br>2 = moderate<br>3 = marked	**Dysplasia**	0 = absent<br>1 = present<br>Comment on cell type and frequency

Supplemental Table S2. Scoring of bone marrow variables.

**Cytology**		**Histology core biopsy**		**Histology postmortem sample**	
**Quality**<br>Particles/slide	0 = 0<br>1 = 1-3<br>2 = 4-9<br>3 = >9	**Quality**<br>Intertrabecular spaces free of artifact	0 = 0-1<br>1 = 2-3<br>2 = 4-5<br>3 = >6	**Quality**<br>Degree of autolysis	0 = marked<br>1 = moderate<br>2 = mild<br>3 = none
**Cellularity**<br>Hematopoietic to adipose tissue	0 = <30%<br>1 = 30-50%<br>2 = 51-70%<br>3 = 71-100%	**Cellularity**<br>Hematopoietic to adipose tissue	0 = <30%<br>1 = 30-50%<br>2 = 51-70%<br>3 = 71-100%	**Cellularity**<br>Hematopoietic to adipose tissue	0 = <30%<br>1 = 30-50%<br>2 = 51-70%<br>3 = 71-100%
**Megakaryocytes**<br>Number per particle	0 = 0-1<br>1 = 2-3<br>2 = 4-9<br>3 = >9	**Megakaryocytes**<br>per 100x field	0 = 0-1<br>1 = 2-3<br>2 = 4-9<br>3 = >9	**Megakaryocytes**<br>per 100x field	0 = 0-1<br>1 = 2-3<br>2 = 4-9<br>3 = >9
**Iron**	0 = absent<br>1 = present<br>2 = abundant	**Iron**	0 = absent<br>1 = present<br>2 = abundant	**Iron**	0 = absent<br>1 = present<br>2 = abundant
**Maturation**	N = normal<br>A = abnormal	**Maturation**	N = normal<br>A = abnormal	**Maturation**	N = normal<br>A = abnormal
**G:E**<br>Granulocytic to erythroid ratio	Calculated or estimated	**G:E**<br>Granulocytic to erythroid ratio	Estimated	**G:E**<br>Granulocytic to erythroid ratio	Estimated
**Blasts**<br>Estimate %	0 = <5%<br>1 = 5-9%<br>2 = 10-19%<br>3 = ≥20%	**Blasts**<br>Estimate %	0 = <5%<br>1 = 5-9%<br>2 = 10-19%<br>3 = ≥20%	**Blasts**<br>Estimate %	0 = <5%<br>1 = 5-9%<br>2 = 10-19%<br>3 = ≥20%
**Lymphocytes or plasma cells**	0 = 0-5%<br>1 = 6-15%<br>2 = 16-30%<br>3 = >30%	**Lymphocytes or plasma cells**	0 = 0-5%<br>1 = 6-15%<br>2 = 16-30%<br>3 = >30%	**Lymphocytes or plasma cells**	0 = 0-5%<br>1 = 6-15%<br>2 = 16-30%<br>3 = >30%
**Dysplasia**<br>Estimate % in erythrocytic, granulocytic and megakaryocytic	0 = none<br>1 = <10%<br>2 = 10-49%<br>3 = >50%	**Dysplasia**<br>Estimate % in erythrocytic, granulocytic and megakaryocytic	0 = none<br>1 = <10%<br>2 = 10-49%<br>3 = >50%	**Dysplasia**<br>Estimate % in erythrocytic, granulocytic and megakaryocytic	0 = none<br>1 = <10%<br>2 = 10-49%<br>3 = >50%
		**Myelofibrosis**	0 = absent<br>1 = present/ suspected	**Myelofibrosis**	0 = absent<br>1 = present/ suspected
**Interpretation**	1 = Not neoplasia<br>2 = MDS<br>3 = AML<br>4 = Other	**Interpretation**	1 = Not neoplasia<br>2 = MDS<br>3 = AML<br>4 = Other	**Interpretation**	1 = Not neoplasia<br>2 = MDS<br>3 = AML<br>4 = Other

Abbreviations: AML, acute myeloid leukemia; MDS, myelodysplastic syndrome

Supplemental Table S3. Details of patient history.

Variable	MDS (42) No.	MDS (42) %	AML (28) No.	AML (28) %
Mean (range) duration of illness in months	4 (0.3 – 24)		3 (0.3 – 24)	
Anorexia	19	45	18	64
Lethargy	27	64	20	71
Vomiting	12	29	6	21
Diarrhea	6	14	5	18
Pica	4	10	0	0
Non-regenerative anemia	30	71	12	43
Thrombocytopenia	12	29	6	21
Neutropenia	7	17	5	18
Leukocytosis	0	0	6	21
Evidence of bleeding[a]	8	19	5	18
Skin lesions, facial swelling, nasal discharge	6	14	3	11
Pyrexia	10	24	14	50
Lymphadenopathy	5	12	8	29
Splenomegaly	2	5	3	11

Abbreviations: AML, acute myeloid leukemia; MDS, myelodysplastic syndrome.
[a]Epistaxis, petechiation, bruising, hematuria, and hematochezia

Supplemental Table S4. Criteria for interpretation of samples as myelodysplastic syndrome (MDS) or acute myeloid leukemia (AML). Persistent anemia, thrombocytopenia, or neutropenia on complete blood cell counts was present in all cases of MDS and in 27/28 cases of AML. The one AML case that did not follow this pattern had an absolute blast count of 8.46 x10^9/L.

Sample type	MDS	AML
**Peripheral blood film**		
Blasts (%)	< 5	0 – 100
**Bone marrow cytology or histology**		
Cellularity (%)	> 70[a]	30 – 100
Blasts (%)	< 20	21 – 100
Dysplastic cells[b] (%)	> 10	0 – 50

[a]8/19 aspirates contained no particles; assumed to be due to myelofibrosis.
[b]Criteria for dysplasia as specified in Materials and Methods.

Supplemental Table S5. White blood cell, neutrophil, blast, and platelet count in dogs with myelodysplastic syndrome (MDS, n=42). Only data that predicted survival in the best fitting Cox regression model were included.

Diagnosis	Case No.	WBC (4.9-15.4 ×10^9/L)[a]	Neutrophils (2.9-10.6 ×10^9/L)	Blasts (0.0-0.0 ×10^9/L)	Platelets (117-418 ×10^9/L)
MDS	1	3	1.9	0	196
	2	8.7	7.1	0	36
	3	30.7	22.1	0.46	424
	4	6.3	3.5	0	314
	5	17	10.7	0	73
	8	0.4	0.1	0	102
	9	0.7	0	0.01	119
	10	1.1	0.4	0.01	50
	11	1.6	1.3	0	7
	12	1.9	0.8	0.02	17
	13[b]	3.6	2.7	0.02	82
	14	3.7	2.5	0	48
	15	3.7	2.3	0.02	26
	16	4	2.7	0.04	631
	17	4.3	3	0	164
	18	4.6	3	0	154
	19	4.8	3.6	0.02	169
	20	5.1	3.2	0	254
	21	5.4	3.6	0	63
	22[b]	5.6	2.6	0	260
	23	5.6	3.4	0	133
	24	6.1	4	0	378
	25	6.4	5	0	640
	26	6.9	5.8	0.03	62
	27	7.1	5.4	0.07	43
	28	7.3	4.2	0	295
	29	7.3	4.3	0	153
	30	8.5	5.4	0	540
	31	8.7	6.3	0	7
	32	9.6	5.2	0	264
	33	10.4	6.5	0	348
	34	11	10.2	0	437
	35	11.2	8.5	0	654
	36	11.2	7.7	0	125
	37	12.2	8.7	0	463
	38	13.1	10.9	0	436
	39	13.2	9.8	0	439
	40	15	12.4	0	309
	41	17.4	16	0	362
	42	24.3	18.9	0	180
	43	31.5	26.1	0	948
	44	36.4	25.6	0	131

[a]Reference interval
[b]Case had two submissions

Supplemental Table S6. White blood cell, neutrophil, blast, and platelet count in dogs with acute myeloid leukemia (AML, n=28). Only data that predicted survival in the best fitting Cox regression model were included.

Diagnosis	Case No.	WBC (4.9-15.4 ×10^9/L)[a]	Neutrophils (2.9-10.6 ×10^9/L)	Blasts (0.0-0.0 ×10^9/L)	Platelets (117-418 ×10^9/L)
AML	6	157.7	20.4	50.46	57
	7	5.6	2.6	1.6	160
	45	1.4	0	0	66
	46	1.4	0.6	0	5
	47	1.8	0.4	0.04	366
	48	2	0	0	21
	49	2.4	1.6	0.06	158
	50	2.7	2.3	0	82
	51	2.9	0.1	0.83	9
	52	3.2	1.7	0	64
	53	4.3	0.1	0.04	183
	54	5.5	2.3	1.32	62
	55	6.1	0.8	0.79	121
	56	7	5.7	0.11	94
	57	7.9	4.1	0.28	79
	58	9	2.1	0.13	148
	59	9.1	7.1	0	192
	60	9.4	5	2.4	39
	61	10.6	7.2	0	16
	62	11.1	8.8	0	44
	63	23.4	14.7	1.63	59
	64	30.8	4.3	13.09	33
	65	31.5	23.8	0.32	89
	66[b]	31.9	25	0	113
	67	34.2	5.3	8.46	181
	68	40.5	30.2	0.81	189
	69	42.3	35.8	0	47
	70	64.7	38.8	2.91	23

[a]Reference interval
[b]Case had two submissions
